# Thoracic MRI in Pediatric Oncology: Feasibility and Image Quality of Post-Contrast Free-Breathing Radial 3D T1 Weighted Imaging

**DOI:** 10.3390/biomedicines13092302

**Published:** 2025-09-19

**Authors:** Patricia Tischendorf, Marc-David Künnemann, Tobias Krähling, Jan Hendrik Lange, Walter Heindel, Laura Beck

**Affiliations:** 1 Clinic for Radiology, University of Münster and University Hospital Münster, Albert-Schweitzer-Campus 1, Building A1, D-48149 Münster, Germany; 2 Department of Anesthesiology, University of Münster and University Hospital Münster, Albert-Schweitzer-Campus 1, Building A1, D-48149 Münster, Germany

**Keywords:** magnetic resonance imaging, infant, children, chest, anesthesia

## Abstract

**Objectives**: To compare the feasibility and image quality of a post-contrast free-breathing radial stack-of-stars 3D T1w turbo-field echo Dixon sequence (3D T1w VANE mDIXON) with a conventional cartesian breath-hold 3D T1w fast-field echo mDIXON sequence in pediatric oncology patients undergoing chest MRI. **Methods**: A total of 48 children (34 females; mean age 5.3 ± 3.7 years) underwent contrast-enhanced chest MRI, with 24 examined using the 3D T1w VANE mDIXON sequence and 24 with a conventional breath-hold 3D T1w mDIXON sequence. Image quality was independently assessed by three radiologists using a 5-point scale. Signal-to-noise ratio (SNR) was measured at two anatomical sites, a homogeneous paraspinal muscle region (SNR_muscle_) and the liver apex (SNR_liver_), while avoiding vessels and signal inhomogeneities. The presence of respiratory artifacts, total imaging time, and the need for general anesthesia or sedation were recorded. Interobserver agreement was determined using Fleiss’s kappa (ϰ), and mean SNR values were compared between groups using an independent samples *t*-test. **Results**: The 3D T1w VANE mDIXON sequence yielded significantly higher SNR_muscle_ and SNR_liver_ (530 ± 120; 570 ± 110 vs. 370 ± 110; 400 ± 90; *p* < 0.001), improved diagnostic image quality by approximately 25%, and reduced respiratory artifacts by about 23%. Interobserver agreement was almost perfect. Importantly, the need for general anesthesia was significantly reduced using the 3D T1w VANE mDIXON (*p* < 0.001). **Conclusions**: Free-breathing 3D T1w VANE mDIXON chest MRI is a feasible and effective imaging approach for pediatric oncology patients, offering superior image quality and reducing the need for general anesthesia compared to conventional methods.

## 1. Introduction

Magnetic resonance imaging (MRI) is a crucial diagnostic tool in pediatric patients, particularly in oncology. Its non-invasive nature, absence of ionizing radiation, and superior soft tissue contrast make it indispensable for the precise and objective evaluation of internal organs [1,2,3]. Early on, MRI was considered suboptimal for lung imaging due to physical limitations, such as the low proton density of pulmonary tissue, large susceptibility differences between tissue and air with signal loss, distortion and signal void artifacts as well as motion artifacts from respiration and cardiac activity. However, ongoing technical advancements have significantly enhanced the utility of thoracic MRI [4,5]. While many of these studies and advancements focus on improving the visualization of the lung parenchyma to establish a diagnostic modality comparable to computed tomography (CT) imaging [6,7,8,9,10], non-contrast-enhanced and contrast-enhanced T1-weighted sequences remain of particular importance in oncological settings, where they are essential for evaluating the potential extent of a tumor and detecting metastatic disease [11,12]. Despite these improvements, performing MRI in young, vulnerable patients remains challenging. Motion artifacts, respiratory-related blurring, the frequent need for general anesthesia, and prolonged acquisition times often compromise both image quality and patient safety. These challenges are especially critical in pediatric oncology, where repeated imaging is often required and diagnostic precision directly influences clinical management.

To mitigate motion and respiratory artifacts, MRI is frequently conducted under general anesthesia. While effective, this approach introduces notable risks, particularly in immunocompromised or critically ill children [13,14]. For awake pediatric patients who are unable to reliably hold their breath for 15–20 s, conventional breath-hold sequences may yield suboptimal or even non-diagnostic results. In such cases, free-breathing techniques provide a promising alternative [6,7]. Notably, contrast-enhanced radial free-breathing T1-weighted imaging has shown distinct advantages over traditional cartesian breath-hold sequences [15,16,17,18]. These radial techniques are more robust to motion and eliminate the need for breath-holding, an important benefit for pediatric oncology patients, who may be physically weakened, fatigued, or too young to follow instructions. Recent studies have demonstrated the superiority of these methods in terms of image quality in pediatric imaging [19,20].

However, data on post-contrast free-breathing radial stack-of-stars T1-weighted sequences in young pediatric patients undergoing thoracic MRI remain limited. Therefore, the aim of this study was to compare the feasibility and image quality of a free-breathing 3D T1w fast field echo sequence (3D T1w VANE mDIXON) with a conventional breath-hold cartesian T1w sequence in pediatric oncology patients.

## 2. Materials and Methods

### 2.1. Study Population

This study received approval from the institutional ethics committee. A retrospective analysis was performed on thoracic MRI scans from a consecutive series of 48 pediatric patients who had undergone either thoracic or thoracoabdominal examinations. These MRI studies were obtained within a two-year interval, ranging from December 2022 to December 2024. Children were separated into two subgroups based on the study period: those examined after the implementation of the 3D T1w VANE mDIXON acquisition (VANE group) and those examined previously with conventional breath-hold sequences (BH group). Furthermore, documentation regarding whether sedation or general anesthesia was required was extracted from the clinical charts.

### 2.2. MR Examinations

All imaging was carried out on a 1.5 Tesla Achieva SmartPath to dStream system (Philips Healthcare, Best, The Netherlands) equipped with a 32-channel dStream Torso array in combination with the built-in 44-channel spine coil. Technical details such as repetition and echo times, slice thickness, interslice gap, flip angle, field of view, and reconstructed voxel size were obtained from the DICOM (Digital Imaging and Communications in Medicine) metadata of each sequence and are summarized in Table 1. For patients in group 1, the routine scan protocol consisted of axial T2w Multi-Vane, coronal T2w SPIR, axial diffusion-weighted imaging, and axial pre- and post-contrast 3D T1w VANE mDIXON. In contrast, the standard protocol for group 2 comprised axial T2w Multi-Vane, coronal T2w SPIR, axial diffusion-weighted imaging and cartesian pre- and post-contrast T1w mDIXON. For every MRI examination, the DICOM files were evaluated, and the time stamps of the first localizer and the final sequence; as additionally, the initiation and completion of contrast-enhanced T1w acquisitions were recorded.

### 2.3. Technical Advantages of the 3D T1w VANE mDIXON Sequence

The 3D T1w VANE mDIXON sequence utilizes a radial stack-of-stars k-space trajectory, combining in-plane radial sampling with slice-wise cartesian encoding along the z-axis. This hybrid approach provides significant motion robustness by distributing respiratory motion artifacts more uniformly across the image, rather than along specific phase-encoding directions as seen in conventional cartesian sequences [21].

Radial sequences intrinsically oversample the center of k-space, which contains the majority of the image’s contrast and signal information. This leads to improved signal averaging and a significantly enhanced signal-to-noise ratio (SNR), which is particularly beneficial in thoracic MRI where tissue proton density is low and physiological motion is high. Additionally, the redundancy in radial trajectories allows for effective motion averaging across respiratory cycles, enabling diagnostic-quality imaging even under free-breathing conditions [15]. In contrast to conventional cartesian sequences, the Vane technique provides 3D volumetric coverage with high spatial resolution, while minimizing the impact of respiratory motion.

### 2.4. Image Quality Data

The evaluation of image quality was performed both subjectively and objectively by three radiologists with different levels of experience: 11 years (PT), 13 years (LB), and 4 years (MK). All reviewers were blinded to patient information and the imaging protocol. Independently, they scored the following aspects on a five-point Likert scale: overall image impression, sharpness, vessel depiction, and stability against respiratory motion. A score of 1 indicated insufficient quality (non-diagnostic global impression, imperceptible edge definition, non-recognizable vessel structures, and severe respiratory motion degradation), whereas 5 indicated excellent image quality (crisp anatomical delineation, no blurring of vessels or parenchymal borders and complete absence of motion artifacts). The criteria used were adapted from Ichikawa et al. [22]. Water-only images served as the basis for the analysis. In addition to Likert scoring, the readers documented whether respiratory motion artifacts were present in each sequence and any pathological findings were recorded. SNR was quantified for post-contrast T1w sequences in the fat-suppressed water-only mDIXON image for both groups, involving the placement of a region of interest (ROI) of approximately 10 mm^2^ over a constant point in a homogeneous region of the paraspinal musculature (SNR_muscle_) at the mid-thoracic level and also in the apex of the liver (SNR_liver_), avoiding vessels and signal inhomogeneities, and determining the mean signal intensity. This site was selected due to its anatomical consistency, low perfusion variability, and minimal susceptibility to motion or flow artifacts. To estimate background noise, a separate ROI was positioned in the air outside the patient’s body, in an artifact free area within the field of view. SNR was calculated as: SNR = mean signal of tissue/standard deviation of background noise [23].

### 2.5. Statistical Methods

Data collection was carried out in Microsoft Excel (Microsoft Corporation) and descriptive statistics were computed. Interobserver reliability of subjective ratings was assessed with Fleiss’ kappa (κ). Interpretation followed the standard convention: κ < 0.20 = slight; 0.21–0.39 = fair; 0.40–0.59 = moderate; 0.60–0.79 = substantial; 0.80–1.00 = almost perfect agreement [24]. To evaluate data distribution, the Kolmogorov–Smirnov test was applied. For normally distributed values, Student’s *t*-test was performed; if variances differed, the Wilcoxon signed-rank test was used instead. A *p*-value < 0.05 was defined as statistically significant. Differences in mean SNR values between the two groups were examined with an independent samples *t*-test. All analyses were run using IBM SPSS Statistics 29.0 (IBM Corporation, Armonk, NY, USA). Data were pseudonymized in line with institutional and legal data protection requirements.

### 2.6. Anesthetic Protocols

Airway management strategies were chosen individually by the responsible anesthesiologist, considering patient condition and the fact that the 3D T1w VANE mDIXON sequence does not require breath-holding. When breath-holding was necessary under general anesthesia, apnea was induced. For sedation, dexmedetomidine combined with propofol was used to maintain spontaneous breathing. In contrast, for general anesthesia, sufentanil and propofol were administered for induction and anesthesia was maintained with sevoflurane.

## 3. Results

### 3.1. Patients’ Characteristics

A total of 48 young children (34 females, 14 males; 5.3 ± 3.7 years) comprised the study population. The most common reason for imaging was neuroblastoma (19/48), followed by lymphoma (7/48) and by rhabdomyosarcoma (6/48). There was no significant difference between both groups (Table 2).

### 3.2. MR Examination, Image Times and Image Quality

As shown in Table 3, the need to repeat post-contrast T1-weighted sequences was lower in the VANE group compared to the BH group (1 vs. 7), reaching statistical significance (*p* = 0.02).

The mean thoracic post-contrast T1w imaging times of all subjects were 155 ± 24 s in the VANE group and 68 ± 29 s for the BH group (*p* < 0.001) while total imaging time in both groups was equal with 41.1 ± 11.2 min vs. 42.7 ± 10.8 min (*p* = 0.4). In the VANE group 16 patients underwent thoracoabdominal MRI vs. 13 patients in the BH group (*p* = 0.37) with an imaging time of 42.4 ± 11.9 min in the VANE group vs. 43.5 ± 10.8 min in the BH group (*p* = 0.69).

**Figure 1:**
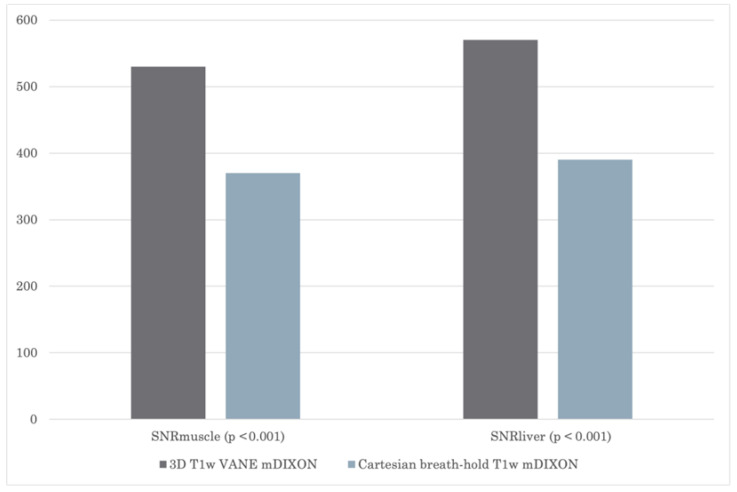
Average data of SNR_muscle_ and SNR_liver_; 3D T1w VANE mDIXON versus cartesian T1w mDIXON sequence.

Using the 3D VANE mDIXON sequence, SNR_muscle_ and SNR_liver_ were significantly higher, with 530 ± 120 and 570 ± 110, compared to the conventional post-contrast exams with breath-hold 370 ± 110 and 400 ± 90 (both *p* < 0.001), Figure 1.

**Figure 2:**
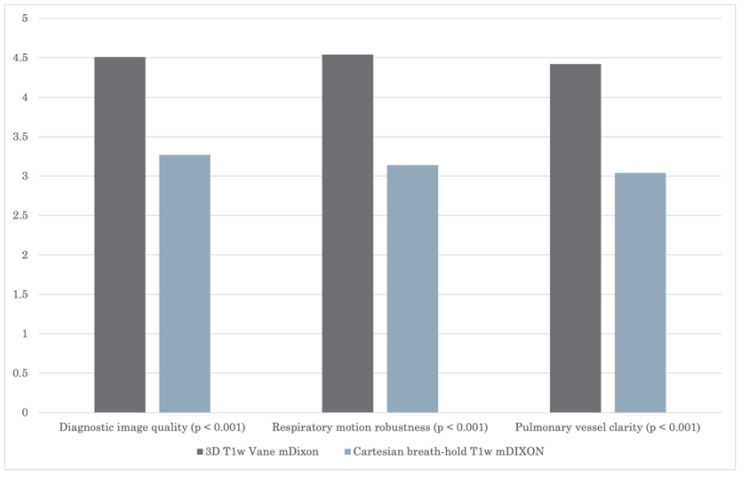
Average subjective diagnostic image quality scores across all readers; 3D T1w VANE mDIXON versus conventional cartesian breath-hold T1w mDIXON sequence.

Subjective image quality was rated in favor for the group examined with the 3D T1 VANE mDIXON sequence. Figure 2 shows the average scores of overall subjective image quality across all readers. The 3D T1 VANE mDIXON sequence significantly improved diagnostic image quality approximately 25% and also reduced respiratory artifacts about 23%, compared to the conventional breath-hold technique.

**Figure 3:**
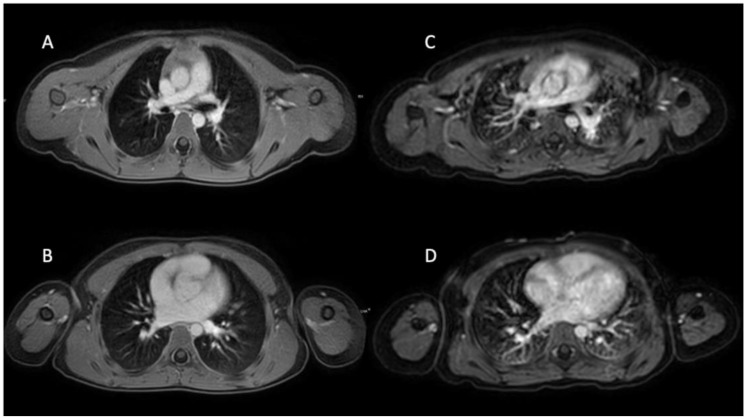
Six-year-old girl with neuroblastoma undergoing 3D T1 VANE mDIXON in sedation (**A**,**B**). Four-year-old girl with rhabdomyosarcoma using conventional cartesian breath-hold T1w mDIXON under general anesthesia (**C**,**D**). Diagnostic image quality ratings on a 5-point Likert scale, with 5 being the best, provided by reader 1 (R1), reader 2 (R2) and reader 3 (R3); (**A**,**B**) R1: 4, R2: 5 and R3: 5, (**C**,**D**) R1: 3, R2: 3 and R3: 4.

According to the results of Fleiss’ kappa analysis, the interobserver agreement ranged from substantial to almost perfect. For the VANE cohort, mean ϰ values across the parameters diagnostic image quality, presence of respiratory artifacts and depiction of pulmonary vessels reached 0.74. In the BH cohort, the corresponding mean ϰ was at 0.85. Detailed tables of reader agreement for each quality criterion are summarized in Table 4. Representative examples illustrating image quality from patients imaged with 3D T1w VANE mDIXON compared to those from the BH group are shown in Figure 3.

### 3.3. Analysis of Anesthetic Protocols

No child in the VANE group required general anesthesia, whereas twelve examinations in the BH group were performed under general anesthesia (*p* < 0.001). The remaining patients were imaged without the use of an artificial airway. Within the VANE group, nineteen children received sedation and five underwent MRI without any anesthetic support. In comparison, in the BH group, two children required sedation and ten were examined without anesthesia (*p* < 0.001 and *p* = 0.12). A detailed overview is provided in Table 2. The imaging time for examinations under general anesthesia was significantly longer compared with sedation or no anesthesia (47.9 ± 10.2 min vs. 40.8 ± 11.6 min vs. 38.8 ± 9.4 min; *p* < 0.001).

## 4. Discussion

In this retrospective cohort study, we evaluated the feasibility and image quality of a post-contrast free-breathing 3D T1w radial stack-of-stars sequence (3D T1w VANE mDIXON) compared to conventional cartesian breath-hold T1w imaging in 48 consecutive young pediatric oncology patients undergoing thoracic MRI. The most frequent indication was neuroblastoma, followed by lymphoma and rhabdomyosarcoma, tumor types for which thoracic involvement and regular follow-up imaging are critical. There were no significant differences in demographic characteristics, clinical indications or in the frequency of thoracic versus thoracoabdominal examinations between the two groups. Although the imaging time for the 3D VANE mDIXON sequence was longer than for the breath-hold technique, the total scan duration did not differ significantly between the groups. This finding is consistent with prior studies [16] and suggests that while the radial sequence requires more time for data acquisition, the overall workflow remains efficient, potentially due to reduced need for repeated sequences, lower incidence of motion-related artifacts and the fact that general anesthesia is usually not required, since no breath-holding is necessary. Importantly, the 3D T1w VANE mDIXON sequence yielded significantly higher SNR_muscle_ and SNR_liver_ values than the cartesian breath-hold sequence. Subjective image quality was also consistently rated superior across all evaluated parameters, including pulmonary vessel clarity and robustness against respiratory motion. Interobserver agreement ranged from substantial to almost perfect, underlining the reliability of image assessment. Diagnostic image quality was markedly improved by about 25% and respiratory artifacts were significantly reduced by approximately 23%. These results are consistent with the prior literature demonstrating that radial free-breathing acquisitions offer superior motion robustness in pediatric and uncooperative patients [17,25,26,27]. This improvement in image quality can be directly attributed to the physical principles of the 3D T1w VANE mDIXON sequence, as outlined in the technical section of this manuscript. The combination of radial in-plane sampling and central k-space oversampling allows for better motion averaging, which results in higher SNR and reduced respiratory artifacts even in free-breathing settings. This underpins the observed diagnostic improvements in our cohort. However, published data specifically evaluating thoracic radial T1-weighted imaging in young pediatric oncology patients remains limited. Our study helps to fill this gap and supports the clinical value of implementing such techniques in routine pediatric thoracic imaging.

Another critical advantage of the 3D T1w VANE mDIXON sequence is its positive impact on anesthesia management. In our study, none of the patients in the free-breathing group required general anesthesia, whereas twelve patients in the breath-hold group did. The elimination of breath-hold requirements allowed more young patients to undergo MRI under sedation or awake, thereby reducing anesthesia-related risks.

This study has several limitations that should be considered. First, the sample size in the relatively young patients, only 48 patients, limits the power of subgroup or rare event analysis. There were no differences in patient characteristics between groups; however, this emphasizes the necessity for ongoing development and validation of advanced techniques where there is not yet a realized advantage for the pediatric patient. Secondly, the single-institution study and the fact that all investigations were performed on a single 1.5 T MRI system from one vendor would inherently tend to limit the applicability of the results. While this ensured technical consistency, the performance of the 3D T1w VANE mDIXON sequence on other scanner types and field strengths may not be fully captured. However, the application of standardized imaging protocols and stable scanner settings support the internal validity of the study.

Additional multicenter validation are recommended to establish the technique’s robustness across different hardware platforms prior to broad clinical implementation. Third, patients in our cohort had a range of malignancies, such as neuroblastoma, lymphoma and rhabdomyosarcoma, likely leading to anatomic and clinical heterogeneity that may affect imaging features as well as anesthesia needs. Nevertheless, the distribution of tumor types was balanced between the two groups, and this heterogeneity reflects clinical practice and may even support broader applicability. Furthermore, patients were assigned to VANE group versus BH group consecutively based on the period of protocol implementation rather than through random allocation, which could introduce bias from unmeasured temporal or clinical factors. A further limitation of our study is the lack of routine summarization of quality-of-life aspects such as patient comfort, anxiety or parental satisfaction. These are especially relevant when discussing the possibility of diminishing the use of general anesthesia or sedation in pediatric imaging and may be worth exploring in future investigations. Moreover, although image quality was assessed using established subjective criteria, the evaluation relied on visual grading by experienced radiologists. While interobserver agreement was high, the lack of an external validation group may limit broader reproducibility. This study focused on feasibility and image quality metrics, such as signal-to-noise ratio and respiratory motion artifacts, rather than diagnostic performance measures like lesion detectability or clinical decision impact. Future studies should investigate how the improved imaging characteristics of the 3D T1w VANE mDIXON sequence translate into diagnostic accuracy and oncologic outcomes. Finally, due to the retrospective nature of the study, we were unable to assess all contributing factors to scan interruptions or prolongation as well as sedation-related complications. However, our results indicate that post-contrast sequences had to be repeated significantly less frequently when using the 3D T1w VANE mDIXON sequence compared to the conventional breath-hold technique.

In conclusion, post-contrast free-breathing 3D T1w VANE mDIXON chest MRI correlates with improved image quality and reduced motion artifacts compared to conventional breath-hold techniques in pediatric oncology, enabling reliable scans without general anesthesia.

## Figures and Tables

**Table 1:** Magnetic resonance imaging (MRI) sequence parameters.

MRI Parameters	3D T1w VANE mDIXON	Cartesian T1w mDIXON
Acq voxel size (mm^3^)	1.35 × 1.35 × 4.00	1.41 × 1.70 × 4.00
Recon voxel size (mm^3^)	0.78 × 0.78 × 2.00	1.2 × 1.2 × 2.00
Field of view (mm)	250	300
Repetition time/echo time 1/echo time 2 (ms)	4.8/1.5/2.6	5.7/1.8/4.0
Flip angle (°)	10	15
Number of averages	1	1
Spacing between acq. slices (mm)	−2	−2
Respiratory motion compensation technique	diaphragm navigated	breath-hold (10 s)
Median scan time (s)	150	60

**Table 2:** Patients’ characteristics. VANE group = patient group using 3D T1w VANE mDIXON sequence; BH group = patient group using conventional T1w sequence with breath-hold.

	All	VANE Group	BH Group	*p* Value
**Patients’ characteristic’s**				
Age (years)	5.3 ± 3.7	4.3 ± 3.1	6.3 ± 4.1	0.05
Female	34	19	15	0.2
Neuroblastoma	19	10	9	0.76
Lymphoma	7	4	3	0.68
Rhabdomyosarcoma	6	2	4	0.16
Tumor lesions findings	20	7	13	0.08
**Anesthetic procedure**				
Sedation	21	19	2	<0.001
General anesthesia	12	0	12	<0.001
No anesthesia	15	5	10	0.12

**Table 3:** MR Examinations and imaging time. VANE group = patient group using 3D T1w VANE mDIXON sequence; BH group = patient group using conventional T1w sequence with breath-hold.

	All	VANE Group	BH Group	*p* Value
**MR Examinations**				
T1 repeating	8	1	7	0.02
Respiratory artifacts	11	0	11	<0.001
Diagnostic image quality	42	24	18	0.009
Thoracoabdominal MRI	29	16	13	0.37
**Imaging Time**				
T1 imaging time (s)	112 ± 50.7	155 ± 23.6	68.2 ± 28.7	<0.001
Examination time (min)	41.9 ± 10.8	41.1 ± 11.3	42.7 ± 10.8	0.4
Examination time (min) thoracic	40.5 ± 10.7	38.6 ± 10.1	41.8 ± 11.4	0.6
Examination time (min) thoracoabdominal	42.9 ± 11.2	42.4 ± 11.9	43.5 ± 10.8	0.69
Examination time (min) general anesthesia	47.9 ± 10.2	-	47.9 ± 10.2	-
Examination time (min) sedation	40.8 ± 11.6	40.7 ± 12.1	41.5 ± 6.4	0.5
Examination time (min) no anesthesia or sedation	38.8 ± 9.4	42.8 ± 8.2	36.8 ± 9.7	0.064

**Table 4:** Image quality ratings for 3D T1w VANE mDIXON and cartesian 3D T1w mDIXON, as evaluated by reader 1 (R1), reader 2 (R2) and reader 3 (R3) on a 5-point Likert scale, with 5 being the best; including Fleiss’s kappa (ϰ).

	3D T1w VANE mDIXON				Cartesian 3D T1w mDIXON			
	R1	R2	R3	ϰ	R1	R2	R3	ϰ
Diagnostic image quality	4.58 ± 0.5	4.46 ± 0.59	4.5 ± 0.51	0.67	3.28 ± 1.06	3.24 ± 1.05	3.3 ± 1.07	0.86
Respiratory motion robustness	4.54 ± 0.51	4.45 ± 0.49	4.64 ± 0.51	0.84	3.12 ± 0.97	3.2 ± 0.91	3.1 ± 1	0.88
Pulmonary vessel clarity	4.42 ± 0.65	4.51 ± 0.65	4.33 ± 0.7	0.71	3.1 ± 0.9	3.08 ± 0.91	2.96 ± 0.84	0.8

## Data Availability

The data presented in this study are available on request from the corresponding author due to privacy policy.

## Abbreviations

BH	Conventional cartesian breath-hold T1w sequence
CT	Computed tomography
DICOM	Digital Imaging and Communications in Medicine
DWI	Diffusion-weighted imaging
MRI	Magnetic resonance imaging
ROI	Region of interest
SNR	Signal-to-noise-ratio
SNR_liver_	Signal-to-noise-ratio of the apex of the liver
SNR_muscle_	Signal-to-noise-ratio of the paraspinal musculature
SPIR	Spectral presaturation with inversion recovery
VANE	Variable Number of Excitations
